# Synthesis and characterization of ZrFe_2_O_4_ and ZrFe_2_O_4_@UiO-66-NH_2_ nanoparticles for efficient immobilization of *Humicola insolens* lipase: a comparative study of precipitation-crosslinking *versus* covalent binding methods

**DOI:** 10.1039/d6na00003g

**Published:** 2026-02-20

**Authors:** Kowsar Azizi, Saba Ghasemi, Ahmad Nikseresht

**Affiliations:** a Department of Chemistry, IL.C., Islamic Azad University Ilam Iran Sb.ghasemi@iau.ac.ir +98-8433351849 +98-8432224827; b Department of Chemistry, Payame Noor University (PNU) P. O. Box 19395-4697 Tehran Iran

## Abstract

This study focused on immobilizing *Humicola insolens* lipase onto magnetic nanoparticles of ZrFe_2_O_4_ and ZrFe_2_O_4_@UiO-66-NH_2_ using precipitation-crosslinking and covalent binding methods. Characterization techniques, including FT-IR, SEM, energy-dispersive X-ray spectroscopy, X-ray diffraction, BET, DLS, and thermogravimetric analysis, confirmed the successful synthesis and functionalization of the supports. Enzyme immobilization was assessed using different buffer systems, and Tris–HCl buffer at a low concentration was chosen as the optimal medium due to its compatibility with the support structure. The precipitation-crosslinking method resulted in enzyme loadings of 260 mg g^−1^ for ZrFe_2_O_4_ and 226 mg g^−1^ for ZrFe_2_O_4_@UiO-66-NH_2_, achieving immobilization efficiencies of about 40% and 60%, respectively. In contrast, the covalent binding technique significantly improved the enzyme loading and immobilization efficiency, with ZrFe_2_O_4_ achieving 305 mg g^−1^ and 65% efficiency. ZrFe_2_O_4_@UiO-66-NH_2_ demonstrated even greater performance, with the immobilization efficiency exceeding 80%. The reusability and thermal stability of the immobilized lipase improved markedly with covalent binding, particularly for the biocatalyst obtained by immobilizing lipase on ZrFe_2_O_4_@UiO-66-NH_2_ nanoparticles. This biocatalyst retained over 70% of its activity after five reuse cycles and retained 40% activity at 80 °C. In contrast, the precipitation-crosslinking method led to a significant decline in activity during successive cycles, with no observable enhancement in enzyme thermal stability using this technique.

## Abstract 2

This study focused on immobilizing *Humicola insolens* lipase onto magnetic nanoparticles of ZrFe_2_O_4_ and ZrFe_2_O_4_@UiO-66-NH_2_ using precipitation-crosslinking and covalent binding methods.

## Graphical abstract

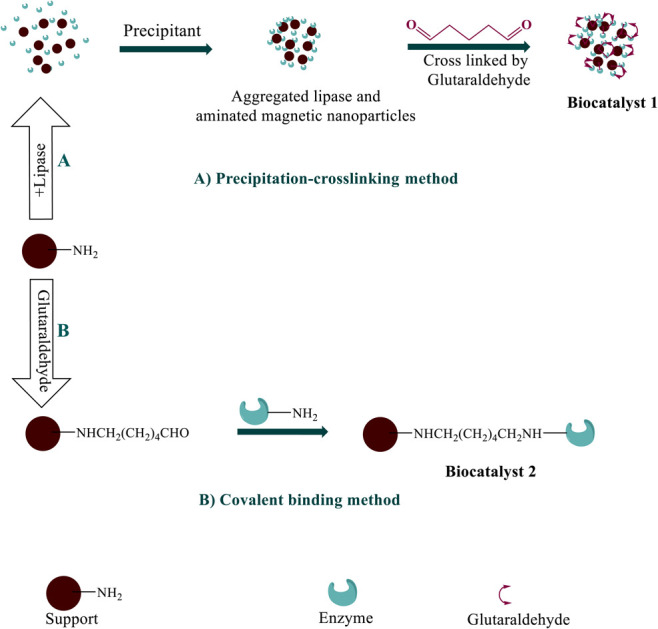

## 1. Introduction

### 1.1. Background on enzymes and their applications

Enzymes are remarkable biological catalysts whose unique properties—such as specificity, efficiency, and the ability to function under mild conditions—make them highly valuable in various fields.^1,2^ One of the most widely utilized enzymes in both scientific research and industrial applications are lipases, which are appreciated for their broad substrate specificity, lack of cofactor requirement, and exceptional stability across various media and conditions.^3,4^ Their versatility allows them to catalyze a diverse array of organic reactions, including hydrolysis, esterification, alcoholysis, acidolysis, interesterification, and amidation.^5,6^ They function through a distinct mechanism called interfacial activation, which enables them to effectively catalyze reactions involving water-insoluble substrates, such as oils.^7^ However, their practical use is often restricted by limitations in stability, reusability, high costs, and the risks of contaminating products with enzymatic residues.^8,9^ Enzyme immobilization, the process of binding enzymes to insoluble support materials, provides an effective way to address these challenges and improves the applicability of enzymes in both industrial and research settings.^8,10^ This method not only overcomes the problems associated with enzyme recovery, but also facilitates downstream processing and continuous operation; in most cases, it improves enzyme properties.^10,11^

### 1.2. Strategies for enzyme immobilization and challenges

Various techniques have been developed for enzyme immobilization, including adsorption, entrapment, encapsulation, covalent attachment, and crosslinking, which utilize physical and chemical means to anchor an enzyme to a support.^12,13^ Based on the nature of the interaction between enzymes and carriers, these methods can be classified into two main categories: reversible and irreversible immobilization techniques.^12,14^ Two of the most widely used techniques for the irreversible immobilization of enzymes are covalent attachment and the cross-linking method.^15^ In the cross-linking method for enzyme immobilization, covalent bonds are formed between enzyme molecules using various cross-linking agents. This process leads to the formation of a stable three-dimensional network of enzymes, which eliminates the need for a carrier material.^15,16^ Although this method is an effective approach for enzyme immobilization, enhancing stability, reusability, and activity under various conditions, there are several limitations associated with it. One significant limitation is low mechanical stability if the crosslinking is not performed appropriately, which can lead to the leaching of the enzyme. In addition, the process of separating cross-linked enzyme aggregates from the reaction mixture for subsequent uses—whether through centrifugation or filtration—can lead to further aggregation. This increase in size results in internal mass transfer limitations, reducing the efficiency of the immobilized enzyme.^17,18^ Various solutions have been proposed to address these challenges and achieve biocatalysts with desirable mechanical properties. One of the most innovative strategies involves the use of magnetic cross-linked enzyme aggregates.^18,19^ In the case of covalent immobilization, a stable covalent bond is formed between the amino acid residues of enzyme molecules and the reactive functional groups that have been introduced onto the surface of the carrier.^20^ This type of irreversible immobilization is a preferred method for attaching enzymes to solid supports, particularly when there is a significant concern about the leaching of the enzyme from the support.^21^ The performance of enzymes that are covalently attached to carriers can be influenced by various factors. These include the characteristics of the carrier, such as its shape, size, and material composition, as well as the method used for the enzyme immobilization to the support and the specific conditions during the coupling process. Therefore, selecting an appropriate immobilization support and technique is essential for creating an effective biocatalyst.^15,22^

### 1.3. Metal–organic frameworks (MOFs) as support materials

One of the most widely used porous and crystalline nanomaterials for enzyme immobilization is metal–organic frameworks (MOFs). MOFs are composed of metal ions or clusters coordinated to organic ligands, forming highly ordered, porous structures with tunable pore sizes and functionalities. Their unique combination of low density, large surface areas, high stability, and structural versatility makes them particularly advantageous for applications such as catalysis, gas storage, separation, and especially enzyme immobilization.^23,24^ Among the large families of MOFs, zirconium-based MOFs (Zr-MOFs) are known for their diverse structural designs and typically exhibit remarkable chemical, thermal, and water stability. They can also preserve their structural integrity when exposed to considerable mechanical stress.^25^ This durability is largely attributed to their strong Zr–O coordination bonds in the framework and robust secondary building units.^26–30^ Consequently, these types of metal–organic frameworks have attracted considerable interest in the field of enzyme immobilization.^31–33^

To further enhance the functionality of these frameworks, magnetic components can be incorporated into their structure, leading to the formation of nanomagnetic MOFs. This is typically achieved by embedding magnetic nanoparticles (*e.g.*, Fe_3_O_4_, CoFe_2_O_4_) within the MOF matrix or growing MOFs on magnetic cores. These nanomagnetic MOFs facilitate easy separation and recovery of immobilized enzymes from reaction mixtures using external magnetic fields, thus enhancing their practical applicability in biocatalysis and other biomedical fields. The structured porosity and functional tunability of nanomagnetic MOFs also allow for tailored interactions with enzymes, improving activity and stability.^34–36^ However, when Zr-based MOFs are used as supports for enzyme immobilization, the choice of an appropriate buffering solution is crucial. Some constituents of buffer solutions may interact with the metal centers or organic linkers of the MOF, leading to leaching of metal ions or structural degradation of the framework.^37,38^

This study specifically focused on the immobilization of *Humicola insolens* lipase onto magnetic nanoparticles of zirconium ferrite and ZrFe_2_O_4_@UiO-66-NH_2_ using two different methods: precipitation-crosslinking and covalent binding. The influence of buffer systems, particularly potassium phosphate and Tris–HCl buffers, on the stability of the UiO-66-NH_2_ framework during the enzyme immobilization process was investigated. Following a thorough characterization of the obtained biocatalysts, the effects of both immobilization techniques on enzyme loading, efficiency, thermal stability, and reusability were elucidated.

## 2. Materials and methods

### 2.1. Materials

*H. insolens* lipase (∼250 U mg^−1^, 170 mg of protein per mL) was purchased from Novozymes situated in Bagsværd, Denmark. ZrOCl_2_·8H_2_O, FeCl_2_·4H_2_O, 2-aminoterephthalic acid (ATA), ZrCl_4_, sodium hydroxide (NaOH, ≥98%), *p*-nitrophenylbutyrate (*p*-NPB), and *N*,*N*-dimethylformamide (DMF, 99.8%) were purchased from Sigma-Aldrich in St. Louis, MO, USA and Merck Chemical Co. in Darmstadt, Germany. All chemicals and solvents used in this study were of analytical grade and utilized as received without any further purification. All FT-IR spectra were recorded on a Bruker Vertex 70 spectrometer. Absorbance measurements were conducted at room temperature employing a PerkinElmer Lambda 25 (USA) UV/Visible spectrometer. The X-ray diffraction analysis was carried out on samples using a STOE Theta–Theta diffractometer (Germany), which was fitted with a Cu Kα (*λ* = 1.5406 Å) radiation source. The system was operated at a voltage of 40 kV and an electrical current of 40 mA. The morphology and topography of the samples used in study were evaluated using a scanning electron microscopy (SEM) system (VEGA3 MIRA II model, Tescan). The thermal gravimetric analysis (TGA) curves of the samples were acquired using a thermogravimetric analyzer (TGA, Shimadzu DTG-60, Japan). Dynamic light scattering (DLS) measurements were performed using a Horiba SZ-100 instrument (Japan) to analyze the particle size and size distribution of the samples. Nitrogen adsorption–desorption measurements were conducted to evaluate the Brunauer–Emmett–Teller (BET) surface area of the samples using a BEL BELSORP MINI II surface area analyzer (Japan). Degassing of the samples was performed with the BEL PREP VAC II system, which provides heating under vacuum up to 450 °C prior to adsorption measurements. Nitrogen adsorption at 77 K was used to determine the BET surface area.

### 2.2. Synthesis of ZrFe_2_O_4_ nanoparticles

Zirconium ferrite magnetic nanoparticles were synthesized using the chemical co-precipitation method, following a previously reported procedure with some modifications.^39^ Initially, 100 mL of distilled water was introduced into a beaker and subjected to a temperature of 80 °C. Subsequently, ZrOCl_2_·8H_2_O (2.48 mmol, 0.8 g) and FeCl_2_·4H_2_O (4.9 mmol, 0.98 g) were incorporated into the solution through vigorous magnetic agitation and under a N_2_ atmosphere. The reaction mixture was adjusted to a temperature of 80 °C and within a pH range of 10–12 by the addition of sodium hydroxide solution (2 M) until the emergence of a black precipitate. The resulting precipitate was subsequently cooled to room temperature, isolated using an external magnet, and subjected to thorough washing with deionized water. Finally, the obtained zirconium ferrite magnetic nanoparticles were dried at 70 °C for 12 h.

#### 2.2.1. Synthesis of ZrFe_2_O_4_@UiO-66-NH_2_

Typically, 0.25 g of the synthesized ZrFe_2_O_4_ nanoparticles were exposed to ultrasonic irradiation for 20 minutes to form a homogeneous dispersion in 30 mL of DMF. Subsequently, 1 mmol (0.233 g) of zirconium(iv) chloride and 1 mmol (0.18 g) of 2-aminoterephthalic acid were introduced into the suspension of magnetic nanoparticles while subjecting it to sonication for a duration of 15 minutes. The reaction mixture was poured into a Teflon-lined autoclave with a volume of 100 mL, which was sealed and kept at 120 °C for 24 h. The container was allowed to cool to room temperature, following which the resulting solid product was collected with a magnet and subsequently washed with ethanol.

### 2.3. Surface modification of the ZrFe_2_O_4_ and ZrFe_2_O_4_@UiO-66-NH_2_ nanoparticles for covalent binding enzyme immobilization

#### 2.3.1. Synthesis of ZrFe_2_O_4_@APTMS nanoparticles

The surface of zirconium ferrite nanoparticles was functionalized with amino groups through a silanization process using 3-aminopropyltriethoxysilane (APTES), following established protocols.^40,41^ Initially, 1 gram of nanoparticles was dispersed in a solution containing 140 mL of ethanol and water in a 1 : 1 ratio by volume. To this suspension, 16 mL of a 25% v/v aqueous solution of ammonium hydroxide was added, and the resulting mixture underwent intense stirring at a temperature of 60 °C for a duration of 24 hours. The black solid product was separated by magnetic decantation and washed with generous volumes of deionized water and ethanol. Following this, the washed nanoparticles were dispersed once again in a solution of ethanol and water (140 mL, 1 : 1 in volume) and subjected to sonication for 30 minutes at room temperature. Next, 1 mL of 3-aminopropyltriethoxysilane (APTES) was added to the suspension, which was then heated to 60 °C under vigorous stirring for 36 hours. The resultant amino-functionalized magnetic nanoparticles were separated magnetically, washed many times with deionized water and ethanol to remove unreacted silane, and dried at room temperature.

#### 2.3.2. Surface modification of ZrFe_2_O_4_@APTMS and ZrFe_2_O_4_@UiO-66-NH_2_ nanoparticles with glutaraldehyde

Following the amino-silanization of ZrFe_2_O_4_ nanoparticles, they were further functionalized with aldehyde groups to enable covalent enzyme binding. For this, 100 mg of the APTES-modified ZrFe_2_O_4_ nanoparticles were dispersed in 18.4 mL of water by sonication for 30 minutes. Subsequently, 1.6 mL of glutaraldehyde (25% v/v) was added to the suspension, and the mixture reaction was agitated at room temperature for 2 hours, allowing the aldehyde groups to attach to the amino-functionalized surface. The magnetically modified support was thoroughly washed with distilled water to remove any unreacted glutaraldehyde and dried overnight at room temperature. According to the same method, the ZrFe_2_O_4_@UiO-66-NH_2_ nanoparticles were modified by a Schiff base reaction between aldehyde groups in glutaraldehyde and amino groups of the linker in UiO-66-NH_2_.

### 2.4. Covalent immobilization of lipase onto the solid supports

The enzyme was covalently immobilized by suspending 10 mg of each modified nanosupport in an Eppendorf tube containing 1 mL of enzyme solution (8.5 mg mL^−1^, 0.01 M buffer at pH 7.4). Subsequently, the suspension was subjected to incubation for a duration of 5 hours at room temperature in a thermoblock shaker at 400 rpm. After the completion of immobilization, the precipitate was magnetically separated and washed with Tris–HCl buffer.

### 2.5. Enzyme immobilization on the supports by the precipitation-crosslinking linkage method

The precipitation-crosslinking method for enzyme immobilization onto the supports of amino silane-functionalized zirconium ferrite and ZrFe_2_O_4_@UiO-66-NH_2_ was performed according to a procedure that had been previously reported.^32,33,42,43^ 20 mg of each nanosupport was dispersed in a tube with 500 µL of the enzyme solution (34 mg mL^−1^, 50 mM buffer at pH 7.4). 2.26 mL of a saturated solution of ammonium sulfate was introduced into the mixture and subsequently agitated at a temperature of 4 °C for a duration of 30 minutes. An appropriate volume of glutaraldehyde (25%) was injected into the above mixture and shaken to facilitate cross-linking of the enzyme molecules to the support material for 2 h. After the incubation time, the supernatant was removed by placing the tube containing the mixture reaction on a magnetic separator. Subsequently, the resulting product was resuspended in Tris–HCl buffer to wash away any residual unreacted enzyme. Protein concentration was estimated based on the calibration curve of bovine serum albumin as the standard protein by the Bradford assay method.^44^ Protein yield was determined as the difference between the total amount of enzyme in the initial solution used for immobilization (*P*_0_) and that in the supernatant (*P*_1_) after filtering of the resulting biocatalyst. This difference was divided by the total amount of enzyme in the solution and then the final result multiplied by 100 according to eqn (1).^45,46^1
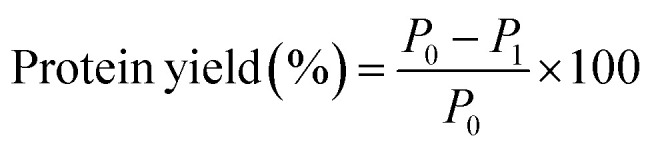


The immobilization efficiency of the immobilized enzyme derivatives was determined using the following formula:^47^2
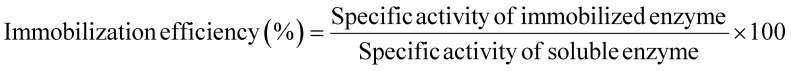


### 2.6. Evaluation of lipase activity assay

The catalytic hydrolysis of *p*-nitrophenyl butyrate (*p*-NPB) by free and immobilized enzymes was evaluated spectrophotometrically to assess their enzymatic performance. Initially, a stock solution of the substrate was freshly prepared by dissolving 4.2 mg of *p*-NPB in 2.5 mL of acetonitrile. 0.5 mL of the stock solution (8 mM) was diluted with 9.5 mL of 10 mM Tris–HCl buffer at pH 7.4 containing 0.1% (v/v) Triton X-100 to yield an assay mixture with a concentration of 0.4 mM. To carry out the enzyme assay, 20 µL of the diluted enzyme sample in buffer was transferred into a 2 mL microcentrifuge tube containing 0.98 mL of the assay mixture. After incubating the reaction mixture at 37 °C for 15 min, the supernatant was used to measure the absorbance at 410 nm. One unit of lipase activity was quantified by measuring the amount of enzyme necessary to liberate 1 µmol of *p*-nitrophenol per minute under standard conditions of the assay method.

### 2.7. Stability parameters

#### 2.7.1. Thermal stability of free and immobilized derivatives of *H. insolens* lipase

The thermal stability of free and immobilized derivatives of enzyme (5 mg) was assessed by pre-incubating them in Tris–HCl buffer at different temperatures (30–80 °C) for 1 h. Subsequently, their residual catalytic activities were immediately determined under standard conditions as described above. In all cases, the highest enzyme activity obtained was assumed to be 100% and used to calculate the relative enzyme activity (%) at other temperatures.

#### 2.7.2. Reusability of immobilized derivatives of *H. insolens* lipase

The reusability of the immobilized enzyme derivatives was further assessed by measuring the recovery of activity over the consecutive hydrolysis reactions of *p*-NPB. Initially, 5 mg of the immobilized biocatalyst was introduced into the assay mixture under the same reaction conditions used to evaluate the hydrolytic activity of the enzyme. Following the first cycle of reaction, the supernatant was separated and subjected to the enzyme activity assay. Afterwards, the recovered biocatalyst was washed several times with Tris–HCl buffer and re-used in the next round enzymatic assay. The relative enzymatic activity of each cycle was calculated as a percentage of the immobilized enzyme activity in the first cycle.

## 3. Results and discussion

In this study, the ZrFe_2_O_4_ and ZrFe_2_O_4_@UiO-66-NH_2_ nanoparticles were synthesized through a stepwise process, as illustrated in Scheme S1. These nanomaterials were chosen as supports for immobilizing *H. insolens* lipase using two methods: precipitation-crosslinking and covalent binding, due to their obvious advantages (Scheme S2).

### 3.1. Characterization of ZrFe_2_O_4_ and ZrFe_2_O_4_@UiO-66-NH_2_ nanoparticles

Magnetic supports were synthesized and subsequently characterized through FT-IR spectroscopy, XRD, SEM, EDX, DLS, and BET techniques. In the FT-IR spectrum of ZrFe_2_O_4_ (Fig. S1a), the peaks observed at 568 cm^−1^ and 453 cm^−1^ could be attributed to Fe–O and Zr–O bands, respectively. The stretching and bending vibration modes of the hydroxyl group were observed at about 3431 and 1627 cm^−1^, indicating the presence of adsorbed water molecules on the support.^39,48,49^ In the case of the ZrFe_2_O_4_@UiO-66-NH_2_ support (Fig. S1b), the symmetric vibrational mode of the primary amine group occurred at 3383 cm^−1^, while the corresponding asymmetric stretch was identified at 3446 cm^−1^. Furthermore, characteristic peaks observed at 769 and 649 cm^−1^ were assigned to the stretching vibrations of the Zr–O bond. The vibration frequency at 1652 cm^−1^ was attributed to the bending vibration of the N–H bond.^43^ The absorption bands associated with the asymmetric and symmetric stretching vibrations of the carboxylate group, as well as the C–N stretching vibrations of aromatic amine, were found at 1571, 1436, 1377, and 1247 cm^−1^, which were in alignment with findings documented in earlier research.^50,51^

**Fig. 1:**
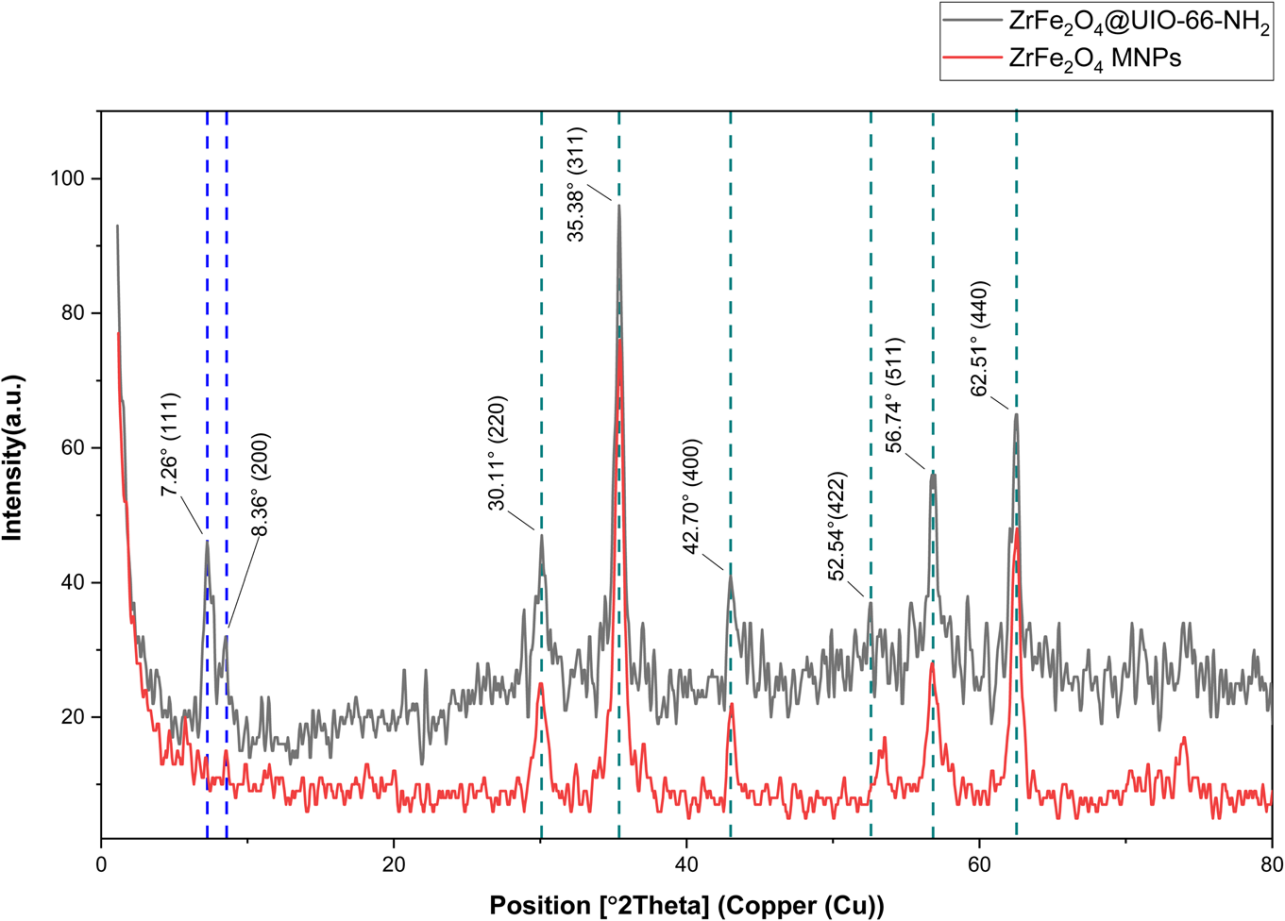
X-ray diffraction patterns of ZrFe_2_O_4_ and ZrFe_2_O_4_@UiO-66-NH_2_ supports.

X-ray diffraction analysis was used to elucidate further details about the structural composition and crystalline characteristics of these materials. Fig. 1 shows the XRD pattern of ZrFe_2_O_4_ and ZrFe_2_O_4_@UiO-66-NH_2_. The diffraction peaks observed at 2*θ* values of 30.11°, 35.38°, 42.70°, 52.54, 56.74°, and 62.51° in the ZrFe_2_O_4_ pattern correspond to the (220), (311), (400), (422), (511), and (440) crystal planes, respectively. These peaks are in good agreement with the literature data,^49^ indicating the successful synthesis of ZrFe_2_O_4_. Zr^4+^ ions occupy both tetrahedral and octahedral interstitial sites in the spinel lattice by substituting Fe^3+^ ions, as previously documented.^49^ The XRD pattern of ZrFe_2_O_4_@UiO-66-NH_2_ clearly displays the characteristic peaks of both ZrFe_2_O_4_ and UiO-66-NH_2_. The appearance of two characteristic peaks at 2*θ* values of 7.26 (111) and 8.36 (200) aligns with the crystalline structure of UiO-66-NH_2_, confirming the successful synthesis and deposition of UiO-66-NH_2_ onto the ZrFe_2_O_4_ nanoparticles.^43^ Notably, the diffraction peaks associated with ZrFe_2_O_4_ remain prominent, indicating that the structural integrity of ZrFe_2_O_4_ is preserved during the synthesis process.

**Fig. 2:**
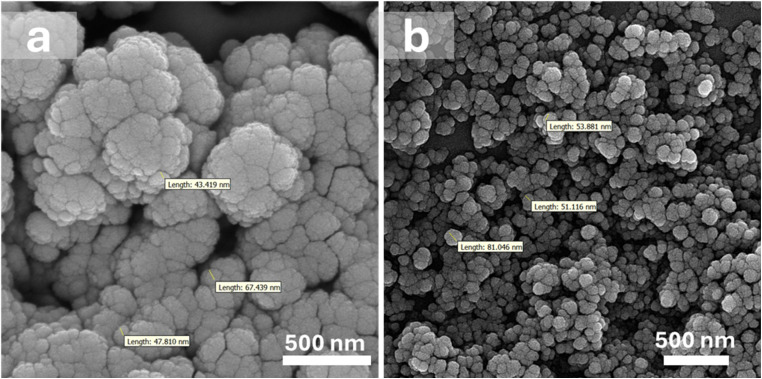
SEM images of (a) ZrFe_2_O_4_ and (b) ZrFe_2_O_4_@UiO-66-NH_2_.

**Fig. 3:**
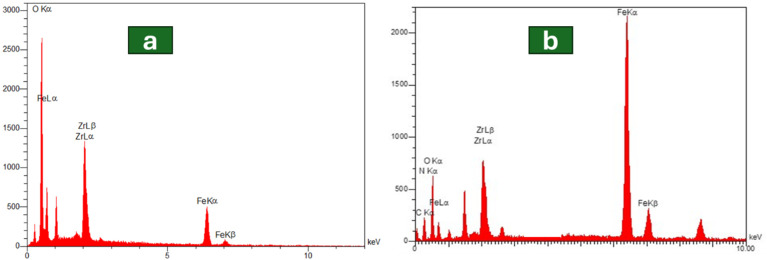
Energy dispersive X-ray spectrum of (a) ZrFe_2_O_4_ and (b) ZrFe_2_O_4_@UiO-66-NH_2_.

The structure of the synthesized supports was further analyzed by SEM (Fig. 2) and EDX (Fig. 3) spectroscopy to provide more information about the composition of the samples and their surface topography. As illustrated in Fig. 2a, the ZrFe_2_O_4_ nanoparticles were formed in a nearly uniform and spherical shape with some degree of agglomeration.^52^ The tendency for agglomeration is common in magnetic nanoparticles due to their strong magnetic dipole–dipole and van der Waals interactions.^53^ Furthermore, the EDX spectrum confirmed the existence of iron (Fe), zirconium (Zr), and oxygen (O) within the ZrFe_2_O_4_ composition (Fig. 3a). Following the coating with UiO-66-NH_2_, the nanoparticles retained their nearly spherical shape, but the extent of agglomeration appeared reduced, and the particles demonstrated a more regular and well-dispersed appearance (Fig. 2b). This improvement in particle dispersion and morphology after coating can be attributed to the stabilizing effect of the MOF layer, which prevents particle aggregation by providing a protective shell that enhances colloidal stability.^54^ Moreover, the detection of carbon and nitrogen peaks in the EDX pattern of ZrFe_2_O_4_@UiO-66-NH_2_ substantiated the successful synthesis UIO-66-NH_2_ on the crystalline magnetic core (Fig. 3b).

The DLS technique was employed to determine the particle size distribution of the synthesized nanoparticle colloidal dispersions. The zirconium ferrite nanoparticles exhibited two distinct populations with mean diameters of 140.8 nm and 741.4 nm, corresponding to smaller particles and slight aggregates, respectively (Fig. S2a). The calculated Z-average hydrodynamic diameter was 316.8 nm with a polydispersity index (PDI) of 0.392, indicating moderate polydispersity. For the ZrFe_2_O_4_@UiO-66-NH_2_ nanocomposite, the *Z*-average hydrodynamic diameter was 310.3 nm with a PDI of 0.462. The main particle population was centered around 211.3 nm, with a minor fraction at 1373.7 nm (Fig. S2b). The slightly smaller hydrodynamic diameter compared to pristine ZrFe_2_O_4_ suggests improved colloidal stability and reduced aggregation due to effective surface functionalization.^54,55^

The particle sizes obtained by DLS were larger than those observed by SEM, consistent with the literature.^55–57^ This difference is attributed to the effect of Brownian motion on the DLS measurements. DLS measures the hydrodynamic diameter including the core, surface layers, and solvation shell, whereas SEM reflects only the solid core size. Consequently, the sizes measured by DLS are generally larger than those observed through electron microscopy techniques.

Nitrogen adsorption–desorption analysis was conducted to evaluate the textural properties of the synthesized materials, and the isotherms were classified according to the IUPAC scheme. The pristine ZrFe_2_O_4_ nanoparticles exhibited a Type II isotherm with very limited nitrogen uptake (Fig. S3), a BET surface area of only 15.2 m^2^ g^−1^, a pore volume of 0.052 cm^3^ g^−1^, and a mean pore diameter of 13.8 nm (Table S1). This pattern reflects the essentially nonporous or macroporous nature of the bare ferrite surface. After modification with UiO-66-NH_2_, the isotherm transformed into a Type I profile, characterized by a steep uptake at low relative pressures, which is typical of microporous frameworks (Fig. S4). Similarly, the surface area increased sharply to 65.5 m^2^ g^−1^ and the pore volume increased to 0.176 cm^3^ g^−1^, while the mean pore diameter decreased to 10.7 nm, confirming the introduction of microporosity by the MOF shell.

### 3.2. Characterization of the prepared biocatalysts

**Fig. 4:**
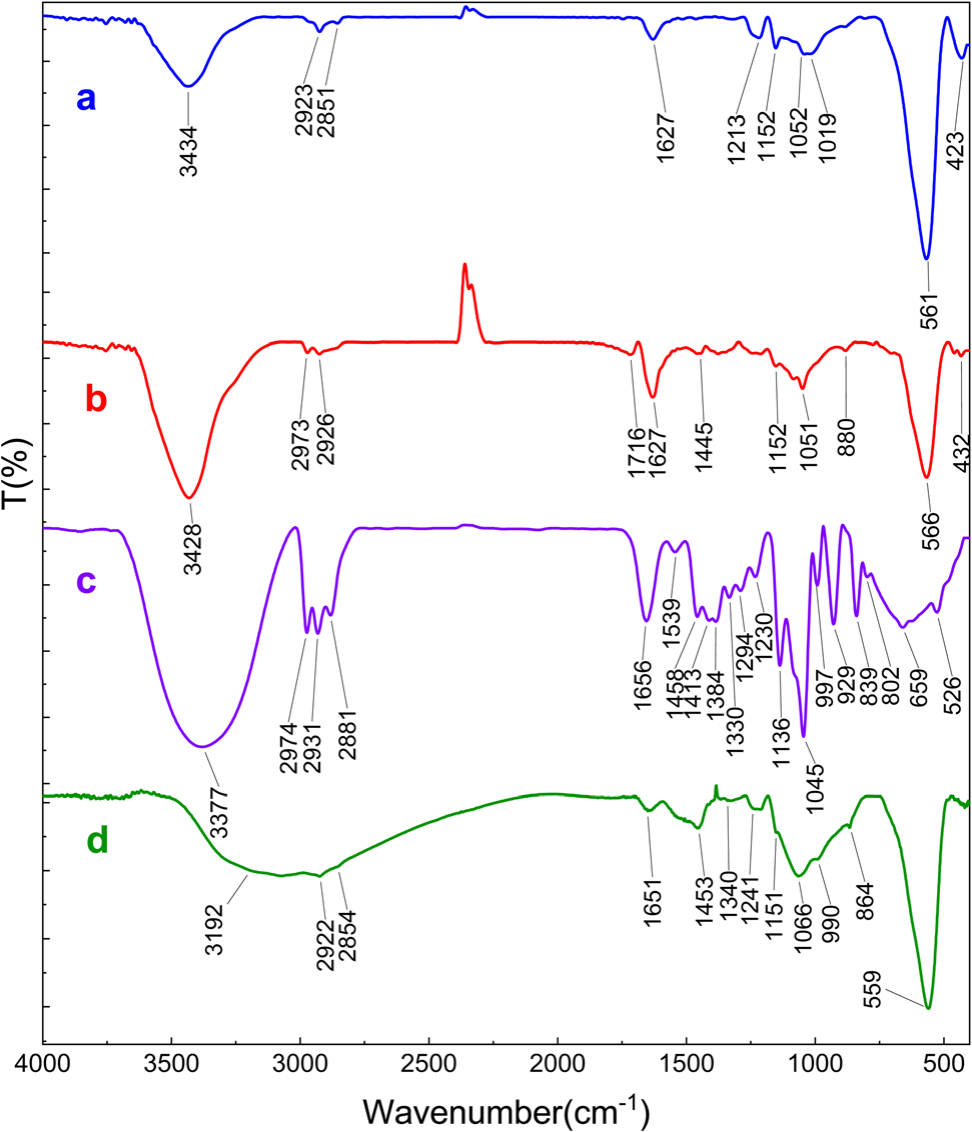
FTIR spectra of (a) ZrFe_2_O_4_ after surface modification with APTES, (b) glutaraldehyde, (c) free enzyme, and (d) immobilized *H. insolens* lipase (HIL) on modified ZrFe_2_O_4_ nanoparticles.

*H. insolens* lipase was subjected to immobilization on the supports of ZrFe_2_O_4_ and ZrFe_2_O_4_@UiO-66-NH_2_ nanoparticles by two distinct methodologies, namely precipitation-crosslinking and covalent binding. The resultant biocatalysts were subsequently characterized through a comprehensive array of techniques including FT-IR spectroscopy, XRD, TGA, SEM, and EDX analysis. To create the covalent attachment of enzymes, the surface of the ZrFe_2_O_4_ nanoparticles was first modified with APTES and then treated with glutaraldehyde. The FT-IR spectra of modified ZrFe_2_O_4_ nanoparticles, free enzyme, and the resulting biocatalyst are shown in Fig. 4a–d. In the spectrum related to APTES-modified nanoparticles (Fig. 4a), the peak observed at 1052 cm^−1^ can be attributed to the stretching vibration of Si–O bonds. Additionally, the absorption bands associated with the asymmetric and symmetric stretching vibrations of the CH_2_ group were detected at 2923 and 2851 cm^−1^, respectively.^40,58^ Following the treatment of the support with glutaraldehyde, the appearance of the new FT-IR absorption band at 1716 cm^−1^ provided evidence that glutaraldehyde has been effectively conjugated to the carrier *via* the amine functional groups (Fig. 4b). The enzyme immobilization on the ZrFe_2_O_4_ nanoparticles was evidenced by the increase in the relative intensity of the spectral band at 1052 cm^−1^, as well as a notable shift in its respective absorption maxima. Furthermore, the appearance of the absorption bands around 1453, 1340, and 1651 cm^−1^ indicated the occurrence of a crosslinking reaction between the enzyme and the functionalized support (Fig. 4d).

**Fig. 5:**
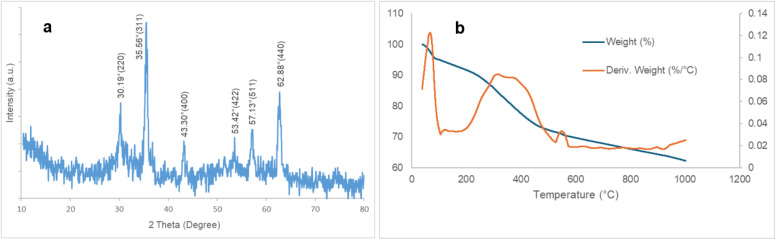
(a) X-ray diffraction pattern and (b) TGA graph of immobilized lipase on activated ZrFe_2_O_4_ nanoparticles.

The characteristic peaks corresponding to the ZrFe_2_O_4_ nanoparticles were similarly detected in the XRD pattern of the biocatalyst, demonstrating that the incorporation of enzyme molecules did not alter the crystalline architecture of the support (Fig. 5a). In addition, thermogravimetric analysis (TGA) was utilized to observe the thermal decomposition process of the prepared biocatalyst (Fig. 5b). The initial reduction in mass detected below 100 °C, approximately 5% (w/w), was associated with the evaporation of physically adsorbed water as well as the partial dehydration of the sample. Subsequently, a gradual mass decrease was observed on the TGA curve up to about 275 °C, followed by a sharper mass loss occurring between 300 and 450 °C. Overall, the observed mass reduction of about 23% within the temperature range of 100–549 °C was ascribed to the thermal degradation of functional moieties and enzyme molecules that were immobilized upon the support.

As evidenced by SEM image (Fig. S5), the boundaries of some particles exhibited a degree of blurriness following the lipase immobilization, which indicated that the chemical reaction taking place during the immobilization process partially changed the surface morphology of the ZrFe_2_O_4_ nanoparticles. The enzyme immobilization on the support was further confirmed by the detection of nitrogen and sulfur spectral peaks in the EDX spectrum corresponding to the biocatalyst (Fig. S6).

**Fig. 6:**
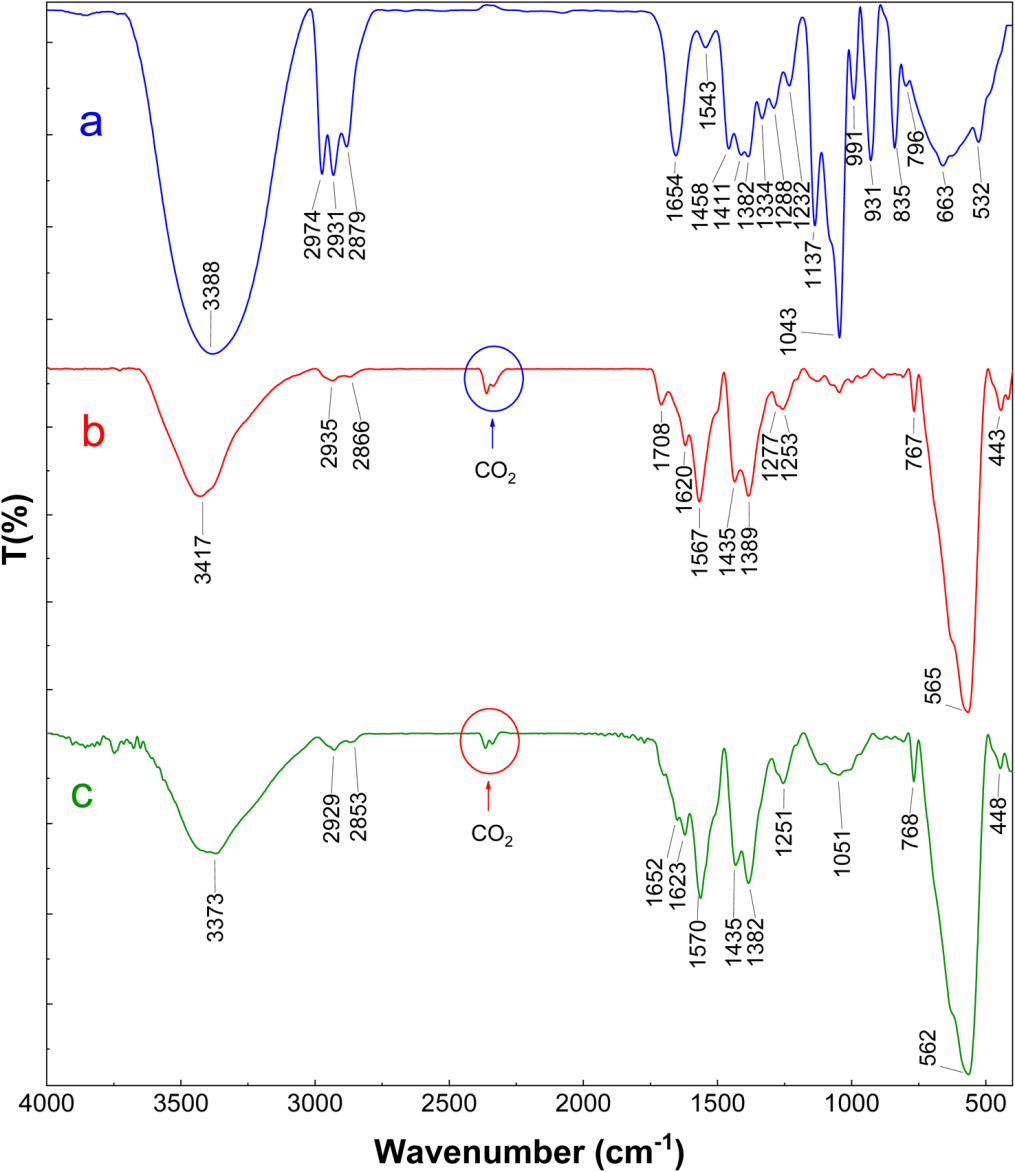
FTIR spectra of (a) free enzyme, (b) glutaraldehyde-functionalized ZrFe_2_O_4_@UiO-66-NH_2_ nanoparticles, and (c) the resulting biocatalyst.

Fig. 6a–c illustrates the infrared spectra corresponding to the *H. insolens* lipase, the glutaraldehyde-functionalized ZrFe_2_O_4_@UiO-66-NH_2_ nanoparticles, and the biocatalyst that was produced as a result. The peak observed at 1708 cm^−1^ provided evidence that glutaraldehyde, functioning as a cross-linking agent for enzyme attachment, was effectively reacted with the amine groups of the carrier (Fig. 6b). Following the process of enzyme immobilization, new characteristic bands emerged at 1051 and 1652 cm^−1^, indicating that the lipase molecules were covalently bonded to the surface of activated ZrFe_2_O_4_@UiO-66-NH_2_ nanoparticles through the cross-linking reaction (Fig. 6c).^32^

**Fig. 7:**
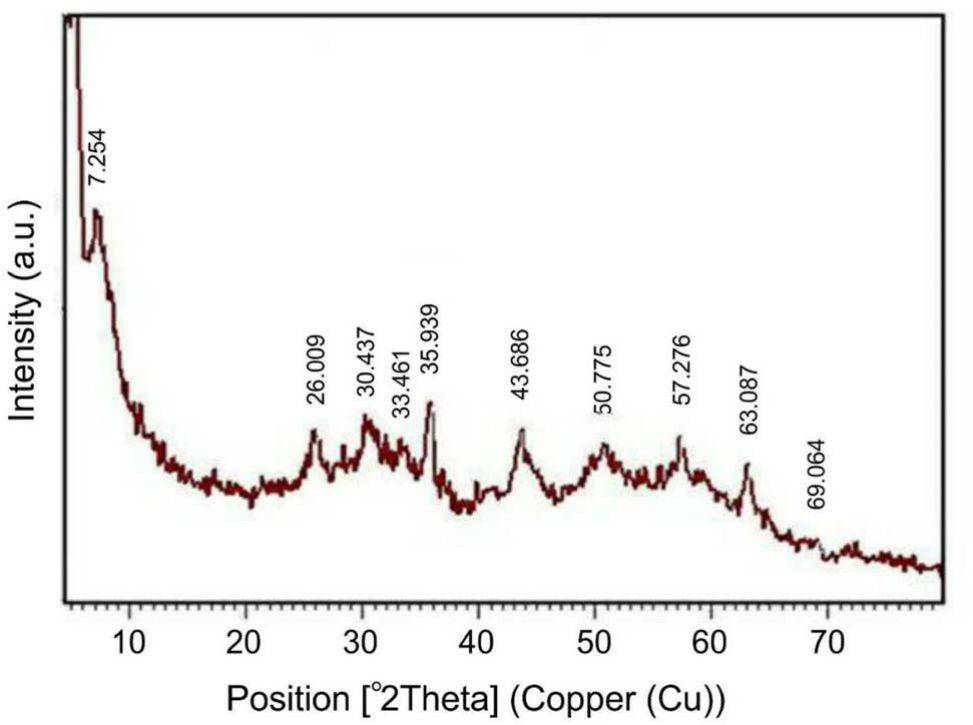
X-ray diffraction pattern of the biocatalyst.

The composition and structural characteristics of the resulting biocatalyst were further elucidated through XRD, SEM, and EDX spectroscopy. Although the position of the peaks in the XRD pattern of the biocatalyst remained consistent, a variation in their intensity was observed, which could be attributed to the interaction between the enzyme and the support (Fig. 7).

**Fig. 8:**
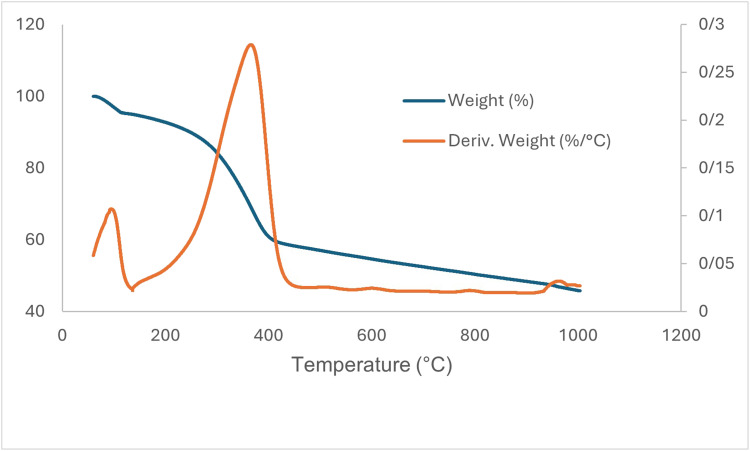
TGA graph of the biocatalyst.

It was further observed from the SEM image (Fig. S7) that the morphological characteristics of the support surface did not significantly change upon enzyme immobilization. The enzyme attachment to the ZrFe_2_O_4_@UiO-66-NH_2_ nanoparticles was finally confirmed by the detection of N and S peaks in the EDX spectrum of the biocatalyst, as illustrated in Fig. S8. The TGA curve of the biocatalyst (Fig. 8) indicated a mass reduction of about 5% at temperatures below 100 °C, which was attributable to the removal of adsorbed water molecules from the support. The rate of weight loss decreased up to about 250 °C, and then accelerated sharply in the temperature range of 250 to 400 °C, correlating with the decomposition of organic components and the enzyme molecules.

### 3.3. The effect of buffer on enzyme immobilization

The composition of buffers can significantly influence the structural stability of MOFs by degrading the coordination bonds. Therefore, a crucial consideration in the utilization of metal–organic frameworks as enzyme supports is their stability in buffered solutions.^59^ Phosphate buffers are commonly used for biological and medical applications, particularly in the field of enzyme immobilization, due to their favorable compatibility with living tissues and microorganisms.^60^ In the present study, enzyme immobilization was initially performed in a 50 mM potassium phosphate buffer. FT-IR analysis of the prepared biocatalyst under these conditions revealed the absence of characteristic MOF peaks and the presence of a prominent absorption at 1022 cm^−1^ (Fig. S9a), which persisted even when the support was exposed solely to phosphate buffer without enzymes (Fig. S9b). This indicates that the observed peak was due to adsorbed phosphate ions rather than successful enzyme immobilization. These observations are in good agreement with the findings reported by Ahmad *et al.*, who investigated the effect of various buffering systems—particularly those containing phosphate ions—on enzyme immobilization within zirconium-based MOFs. Through detailed spectroscopic analyses, they demonstrated that the absorption band observed around 1000 cm^−1^ in the FT-IR spectra of enzyme/MOF composites originates from adsorbed phosphate ions rather than from successful enzyme immobilization. Their results further revealed that phosphate ions interfere with enzyme loading by blocking the MOF pores and disrupting MOF stability.^38^

To further assess the impact of phosphate buffer on the MOF structure, XRD analysis was conducted on the support after immersion in phosphate buffer. The XRD pattern showed a significant loss of the characteristic peaks of ZrFe_2_O_4_@UiO-66-NH_2_, with the disappearance of the main diffraction peaks and a notable reduction in crystallinity (Fig. S10). These results confirm that phosphate buffer induces substantial degradation of the MOF structure. On the other hand, the main peaks of both the support and the enzyme were distinctly detected in the FT-IR spectrum of the biocatalyst prepared with low concentrations of Tris–HCl buffer (≤10 mM). This indicates that the low-concentration of Tris–HCl buffer was significantly compatible with the UiO-66-NH_2_ structure. Similar results were obtained by Bůžek *et al.* when they investigated the stability of the UiO-66 framework in commonly used buffer solutions.^37^ Phosphate buffers led to the degradation of the UiO-66 structure; therefore, their use should be avoided at any concentration. In contrast, low concentrations of TRIS buffer have been introduced as an appropriate medium for UiO-66. In phosphate buffer solutions, phosphate ions behave as ligands capable of replacing organic linkers in MOFs. These ions significantly reduce the stability of metal–organic frameworks, particularly those structured with carboxylate ligands and high-valent metal ions.^61^ Therefore, a low concentration of Tris–HCl buffer was used for enzyme immobilization in the present research due to its compatibility with the support structure.

### 3.4. Immobilization yield and efficiency of the prepared biocatalysts by the precipitation-crosslinking method

The Bradford protein assay determined the total amount of enzyme attached to the supports, while the residual activity of immobilized lipase was assessed by the activity assay. The precipitation-crosslinking method resulted in the enzyme loading of 260 mg g^−1^ on ZrFe_2_O_4_ nanoparticles, achieving an immobilization efficiency of about 40%. With an estimated immobilization efficiency of around 60%, the enzyme loading onto ZrFe_2_O_4_@UiO-66-NH_2_ nanoparticles was calculated to be 226 mg g^−1^ of the support. In the case of both supports, the effect of cross-linking time on the immobilization yield and the enzymatic activity of the immobilized *H. insolens* was also investigated. As can be seen in Fig. S11, the cross-linking time significantly affected the yield of immobilization as well as the catalytic efficacy of the immobilized lipase on the ZrFe_2_O_4_@UiO-66-NH_2_ support. By increasing the coupling time to 2 hours, the highest relative catalytic activity was achieved. In contrast, a reduction in the enzymatic activity was identified when the cross-linking time was extended beyond 2 h. Similar behavior was reported by Cao *et al.*^43^ for soybean epoxide hydrolase immobilization onto the nano-/microscale UiO-66-NH_2_ MOF. This could be due to the excessive covalent interactions occurring between the active groups situated on the support's surface and the amino groups of the enzyme.^62^ It has been already documented that lipase's maximum activity can be attained when its active site amino acids do not interact with the support.^15^ The immobilization yield followed a similar trend, peaking after two hours and subsequently remaining nearly constant with further increases in time. The same results were achieved using zirconium ferrite as the enzyme immobilization support. Therefore, it was found that a cross-linking time of 2 h proved to be optimal for the successful immobilization of *H. insolens* onto both supports using the precipitation-crosslinking method. A typical example of enzyme immobilization by the precipitation-crosslinking method onto a support is evidenced in the research conducted by Chen *et al.*,^33^ who successfully immobilized porcine pancreatic lipase onto UiO-66-NH_2_ with a protein loading capacity of 98.31 mg g^−1^. Cao *et al.*^42^ used the same method to immobilize papain on magnetic nanocrystalline cellulose. In this study, the support was first activated by l-cysteine and subsequently conjugated to the enzyme.

Compared to the precipitation-crosslinking method, covalent binding immobilization resulted in a considerable improvement in enzyme loading and immobilization efficiency in the case of both supports. A total of 305 mg of the enzyme was loaded per gram of ZrFe_2_O_4_ nanoparticles, yielding an efficiency of about 65%. Furthermore, the efficacy of lipase immobilization on the ZrFe_2_O_4_@UiO-66-NH_2_ support was enhanced to over 80% by the covalent bonding technique (Table S2). The percentage of immobilized lipase increased significantly by extending the coupling time to 5 hours and then exhibited a relatively stable trend. Consequently, a cross-linking time of 5 hours was determined to be optimal for the covalent immobilization of the enzyme on both supports.

### 3.5. Reusability of immobilized derivatives of enzymes

By immobilizing enzymes, they can be easily separated from the reaction mixture and utilized repeatedly, enhancing their overall efficiency and minimizing waste. This feature makes them particularly valuable in industrial processes and biocatalysis.^18^ Therefore, the immobilized biocatalysts were subjected to multiple applications to evaluate their effectiveness in recovering the reaction medium.

**Fig. 9:**
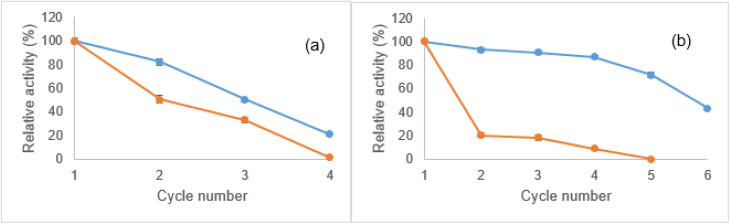
Reusability of the immobilized enzyme on (a) ZrFe_2_O_4_ and (b) ZrFe_2_O_4_@UiO-66-NH_2_ nanoparticles using precipitation-crosslinking (
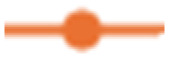
) and covalent binding (
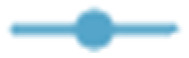
) methods, measured with *p*-NPB. Activities are shown as percentages of initial activity (set at 100%) over multiple cycles, with data as mean ± SD (*n* = 3).

The reusability potential of the enzyme immobilized on zirconium ferrite nanoparticles using both immobilization methods is depicted in Fig. 9a. As indicated, the covalent bonding technique yielded a biocatalyst with better repeatability compared to the precipitation-crosslinking method. The biocatalyst derived from the precipitation-crosslinking method exhibited a reduction of about 50% of its initial catalytic activity following two operational cycles and became entirely inactive by the fourth cycle. On the other hand, the enzyme immobilized through the covalent approach retained 80% of its activity after two uses, with its activity decreasing to 50% after the third cycle. As a result of the covalent bonding of the enzyme to the ZrFe_2_O_4_@UiO-66-NH_2_ support, a significant improvement in the reusability of the biocatalyst was achieved, allowing it to retain over 70% of its initial activity after five reuse cycles. This can be attributed to the formation of strong covalent bonds between the enzyme and the support, which significantly prevents enzyme leakage and consequently enhances the reusability and performance of the immobilized enzyme in various applications.^63^ Conversely, the results related to the reusability of the biocatalyst prepared by the precipitation-crosslinking method on this support were unsatisfactory, as an 80% loss of activity was observed between the first and second catalytic reactions (Fig. 9b). The drastic decrease in residual activity of the enzyme immobilized on both supports using the precipitation-crosslinking method over the consecutive cycles could be attributed to the enzyme leaching from the supports under the reaction conditions.^64^ Hojnik Podrepšek *et al.*^18^ successfully achieved the immobilization of transglutaminase onto iron magnetic nanoparticles using a method comparable to that described in this study. Initially, the enzyme molecules were precipitated with a precipitating agent, followed by cross-linking to amino silane-functionalized magnetic nanoparticles using glutaraldehyde as the cross-linking agent. There was a significant reduction, approximately around 90%, in the residual activity of the immobilized transglutaminase after just three catalytic rounds. The observed reduction in the activity of the immobilized enzyme was primarily attributed to the mechanical loss of enzyme during the washing and centrifugation processes. These processes lead to the loss of aggregates that are not recoverable, consequently affecting the overall enzyme activity.^18^ In the study conducted by Perzon and colleagues, three different types of precipitants (ammonium sulfate, polyethylene glycol, and *tert*-butyl alcohol) were used to prepare cross-linked enzyme aggregates of cellulase. The type of precipitant was found to significantly influence the reusability of the immobilized enzyme. In contrast to polyethylene glycol-CLEA, which demonstrated a retention of 40% of its initial activity after four cycles, the ammonium sulfate-CLEA retained only 10% of its activity after just one cycle.^65^

### 3.6. Thermal stability of free and immobilized enzymes

Enhancing the thermal stability of enzymes is critical for their efficacy, especially in industrial applications that involve high-temperature processes.^66^ Scientific research has demonstrated that the thermal stability of enzymes can be improved through immobilization.^67,68^

**Fig. 10:**
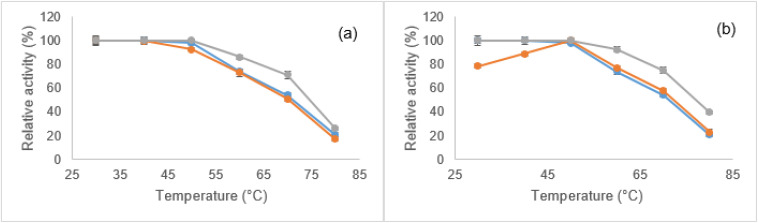
Effect of temperature on the relative activity of free (
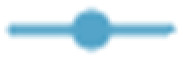
) and immobilized *H. insolens* lipase (HIL) on (a) ZrFe_2_O_4_ and (b) ZrFe_2_O_4_@UiO-66-NH_2_ nanoparticles by two methods of precipitation-crosslinking (
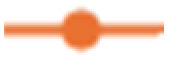
) and covalent binding (
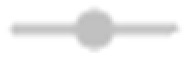
). Activities are shown as a percentage of the maximum (100%), with data as mean ± SD (*n* = 3).

In the current research, the effect of immobilization on the thermal stability of the enzyme was evaluated by subjecting both the soluble form and immobilized derivatives of lipase to a temperature range of 30 to 80 °C. As illustrated in Fig. 10, the free enzyme begins to lose its activity at temperatures above 50 °C, with a more pronounced decline occurring after 60 °C. By the time the temperature reaches 80 °C, the enzyme has lost about 80% of its initial activity, indicating significant thermal denaturation or inactivation. The thermal stability of the *H. insolens* lipase was enhanced by its covalent attachment to both synthesized supports, especially when it was bound to ZrFe_2_O_4_@UiO-66-NH_2_ nanoparticles. For instance, the enzyme immobilized on the zirconium ferrite support retained about 25% of its original activity at 80 °C, in contrast to the ZrFe_2_O_4_@UiO-66-NH_2_ which preserved 40%.

The enhanced thermal stability could be attributed to strong covalent interactions between the enzyme and the support. These interactions may prevent enzyme detachment and denaturation at higher temperatures.^11,69,70^ Furthermore, the integration of the magnetic phase of ZrFe_2_O_4_ into the well-known metal–organic framework UiO-66-NH_2_ has resulted in the formation of the ZrFe_2_O_4_@UiO-66-NH_2_ support, which benefits from the advantages of both components. The functional groups present in UiO-66-NH_2_ facilitate specific interactions between the composite and the enzyme, thereby improving the thermal stability of the resulting biocatalyst.^71^

As mentioned in the Introduction section, MOFs are highly valued as supports for enzyme immobilization due to their tunable organic linkers and intrinsic void spaces, which facilitate guest–host interactions. While traditional approaches often focus on encapsulating enzymes within the MOF pores, this can be limited by the size and stability constraints of large organic linkers, as well as synthesis challenges and cost considerations.^72^ Recently, the surface chemistry of MOFs has gained attention as a promising alternative for enzyme immobilization. Introducing functional groups such as amino (–NH_2_) groups into the MOF framework, as exemplified by UiO-66-NH_2_, significantly enhances the interaction between the enzyme and the support. These amino groups serve as active sites for covalent binding, especially when used with cross-linkers like glutaraldehyde. Glutaraldehyde reacts specifically with primary amines to form stable Schiff base linkages, resulting in the robust covalent attachment of enzymes and improved immobilization efficiency.^73^ The incorporation of amino functionalities also modifies the surface properties of the MOF, including its acid–base characteristics and overall surface chemistry, as demonstrated in the literature.^74–76^ Such modifications can lead to an increase in surface defects, such as missing linkers and clusters, that create additional active sites. These defects facilitate stronger interactions with enzyme molecules through hydrogen bonding and electrostatic interactions, thereby promoting better enzyme anchoring and orientation.^74^ Furthermore, amino groups can influence the electronic environment of the framework surrounding the enzyme, reducing conformational flexibility and enhancing thermal stability.

The thermal stability of the biocatalyst resulting from the immobilization of *H. insolens* lipase on ZrFe_2_O_4_@UiO-66-NH_2_ support through the precipitation-crosslinking method exhibited a slight improvement compared to that of the free enzyme. However, the process of attaching the enzyme to the zirconium ferrite nanoparticles by this method did not result in any enhancement of its thermal stability.

Although immobilization offers benefits like easier separation and possible reusability, it does not necessarily ensure enhanced stability for every enzyme.^77^ The thermal stability of immobilized enzymes can be similar to that of free enzymes, particularly when the immobilization is achieved through physical or ionic adsorption. This is primarily due to the nature of the reversible linkages formed between the enzyme and the support material.^64,78^ For instance, the study by Coutinho and colleagues revealed significant differences in the thermal stability of three enzymes—phytase, xylanase, and β-glucosidase— when they were immobilized on the magnetic hydroxyapatite/CoFe_2_O_4_ nanoparticles. It was found that β-glucosidase exhibited enhanced thermal stability after immobilization, which could be beneficial for its industrial applications. In contrast, the immobilization of phytase and xylanase did not yield any improvements in thermal stability relative to their free forms.^64^ The effectiveness of immobilization in enhancing enzyme stability is significantly influenced by various factors, such as the technique used for immobilization, the characteristics of the carrier matrix, the location and quantity of binding points, the conformational flexibility of the matrix, the properties of the spacer used, and the specific conditions of the immobilization process.^77,79^

## 4. Conclusion

In this study, two different immobilization strategies, precipitation-crosslinking and covalent binding, were employed and compared for the immobilization of *H. insolens* lipase onto the magnetic nanoparticles of zirconium ferrite and ZrFe_2_O_4_@UiO-66-NH_2_. Characterization techniques validated the successful synthesis and functionalization of both supports, confirming their structural integrity and functional capabilities. The findings revealed that covalent binding significantly enhanced both enzyme loading and immobilization efficiency compared to the precipitation-crosslinking method, with a maximum enzyme loading of 305 mg g^−1^ for ZrFe_2_O_4_ and over 80% efficiency for ZrFe_2_O_4_@UiO-66-NH_2_. The study also emphasized the importance of buffer composition in enzyme immobilization, with low concentrations of Tris–HCl being optimal for preserving the structural integrity of the UiO-66-NH_2_. Enzyme immobilization by the covalent bonding method demonstrated a notable advantage in terms of repeatability and thermal stability. This improvement was particularly significant when ZrFe_2_O_4_@UiO-66-NH_2_ nanoparticles were used as the support. In contrast, the precipitation-crosslinking method resulted in a significant decline in activity upon successive uses.

Overall, the improved thermal stability and reusability of lipase immobilized on ZrFe_2_O_4_@UiO-66-NH_2_ nanoparticles make this biocatalyst a promising candidate for various industrial applications. Additionally, these findings open avenues for future research focused on refining enzyme immobilization techniques to further improve biocatalytic efficiency.

## Author contributions

Kowsar Azizi: writing the original draft, laboratory experiments, characterization of the catalysts, software and writing of the manuscript draft. Saba Ghasemi: supervision, conceptualization, analysis, review and editing of the final version and submission of the manuscript for publication. Ahmad Nikseresht: providing key chemical materials and instruments essential for the research, conceptualization, and review and editing of the final version.

## Conflicts of interest

The authors declare that they have no competing interests.

## Supplementary Material

NA-008-D6NA00003G-s001

## Data Availability

The authors declare that all data in this manuscript are available upon request. Supplementary information (SI): characterization data of the nanoparticles and biocatalysts, synthesis schemes (S1 and S2), analytical results (Fig. S1–S10, Table 1), cross-linking time effect on enzyme activity (Fig. S11), and immobilization data (Table 2). See DOI: https://doi.org/10.1039/d6na00003g.
