# Supplementary material for: Synthesis and characterization of ZrFe2O4 and ZrFe2O4@UiO-66-NH2 nanoparticles for efficient immobilization of Humicola insolens lipase: a comparative study of precipitation-crosslinking versus covalent binding methods
Source: Nanoscale Adv. 2026 Feb 20;8(6):2043–56. doi: 10.1039/d6na00003g (PMC12923072; doi:10.1039/d6na00003g)
Supplement: NA-008-D6NA00003G-s001 [file NA-008-D6NA00003G-s001.pdf]

Supplementary Information (SI) for Nanoscale Advances.
This journal is © The Royal Society of Chemistry 2026

Synthesis and characterization of $ZrFe_2O_4$ and $ZrFe_2O_4$@UiO-66-NH_2 nanoparticles for efficient immobilization of *Humicola insolens* lipase: a comparative study of precipitation-crosslinking versus covalent binding methods

Kowsar Azizi[a], Saba Ghasemi[a,*], and Ahmad Nikseresht[b]

[a]Department of Chemistry, Il.C., Islamic Azad University, Ilam, Iran.

[b]Department of Chemistry, Payame Noor University (PNU), P.OBox 19395-4697, Tehran, Iran.

[*]Corresponding author *E-mail address*: Sb.ghasemi@iau.ac.ir (S. Ghasemi), Tel: (+98-8432224827), Fax: (+98-8433351849)

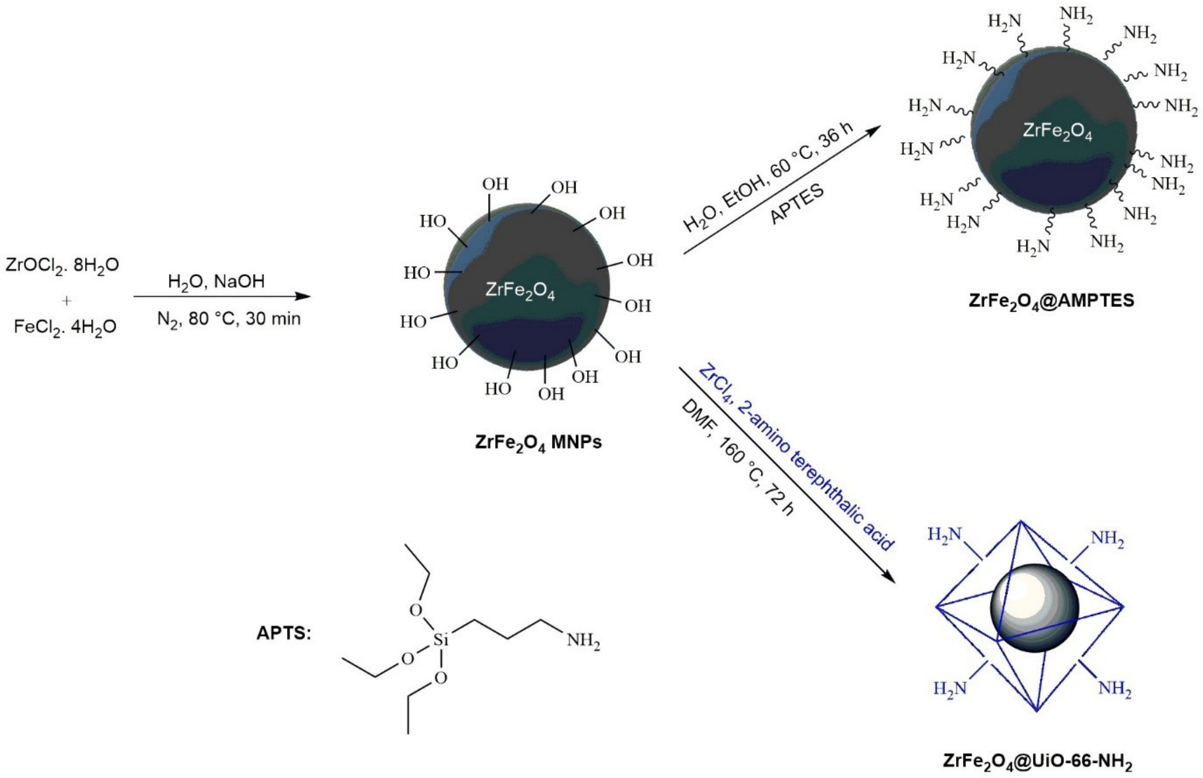

Scheme S1. Schematic representation of the stepwise synthesis pathways for the preparation of $ZrFe_2O_4$ and $ZrFe_2O_4$@UiO-66-NH_2 nanoparticles.

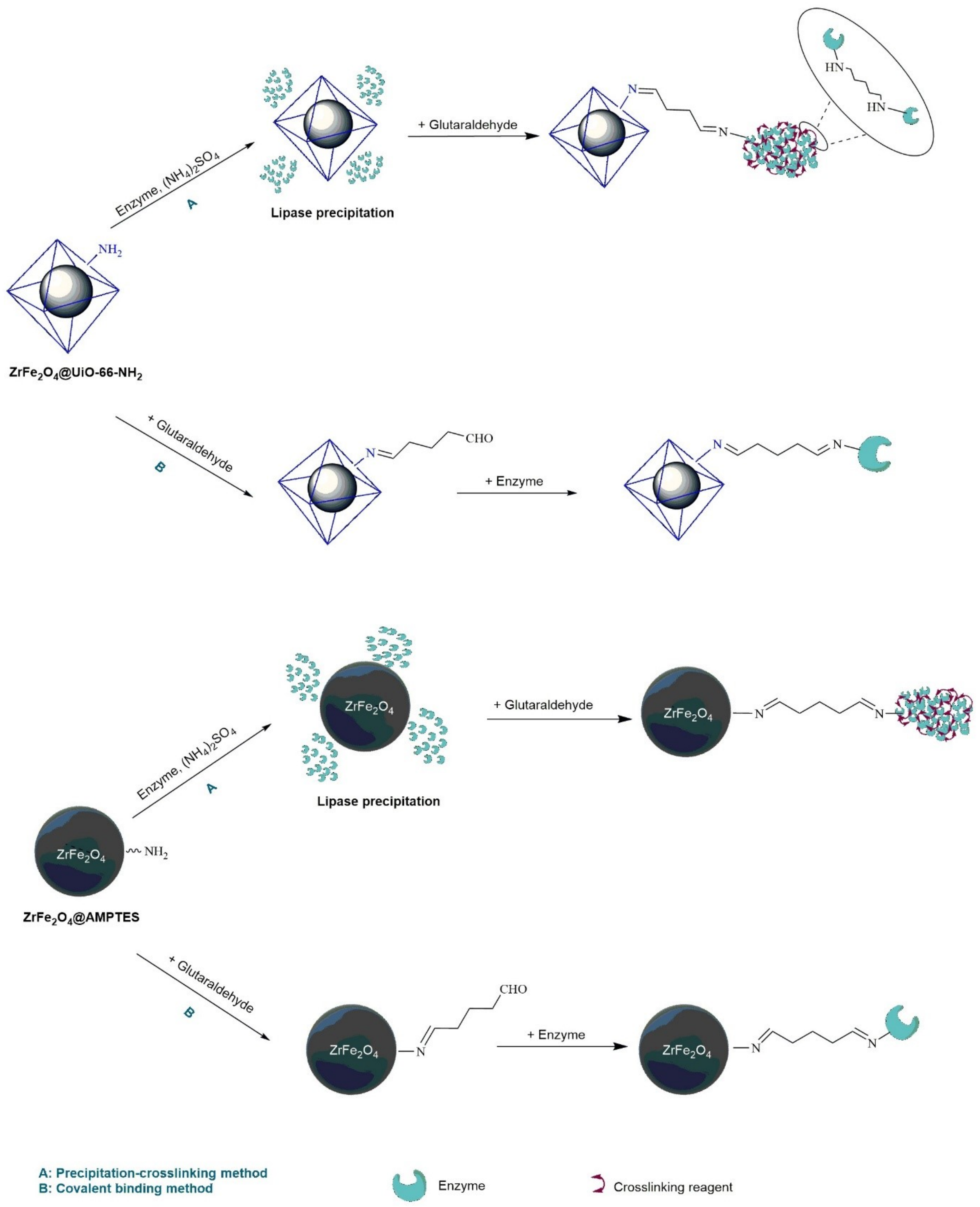

Scheme S2. Schematic illustration of enzyme immobilization on the $ZrFe_2O_4$ and $ZrFe_2O_4$@UiO-66-NH_2 nanoparticles via (A) precipitation-crosslinking and (B) covalent binding methods.

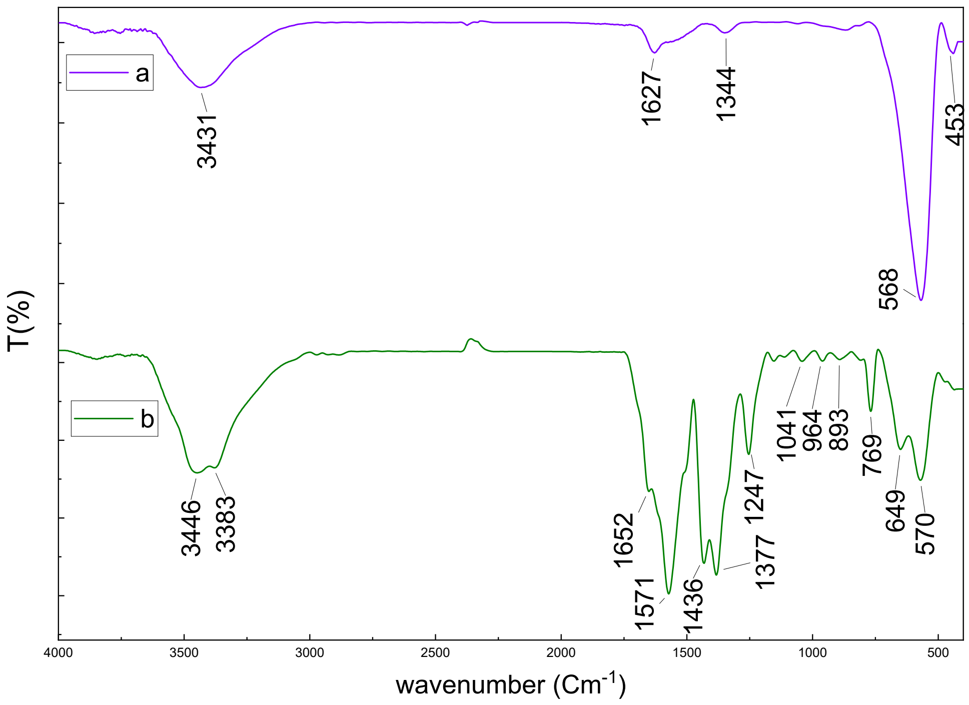

Fig. S1 FTIR spectra of (a) $ZrFe_2O_4$ and (b) $ZrFe_2O_4$@UiO-66-NH_2.

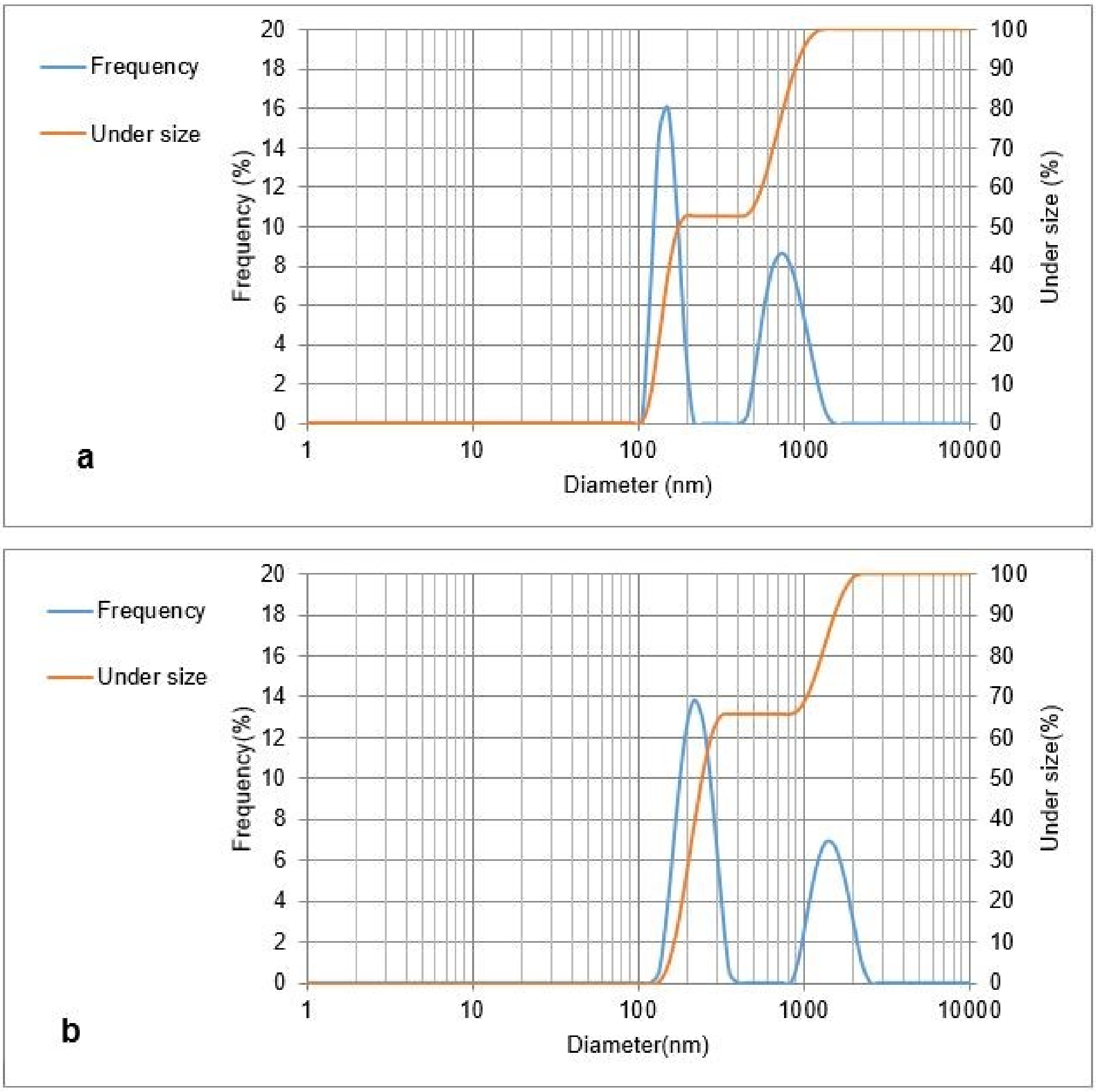

Fig. S2 Size distribution of (a) $ZrFe_2O_4$ and (b) $ZrFe_2O_4$@UiO-66-NH_2 nanoparticles as studied by DLS.

Table 1. BET textural parameters of prepared magnetic nanoparticles.

Sample	BET surface area ($m^2 \cdot g^{-1}$)	Pore volume ($cm^3 \cdot g^{-1}$)	Mean pore diameter (nm)	Isotherm type (IUPAC)
$ZrFe_2O_4$	15.2	0.052	13.8	Type II (nonporous/macroporous)
$ZrFe_2O_4$@UiO-66-NH_2	65.5	0.176	10.7	Type I (microporous)

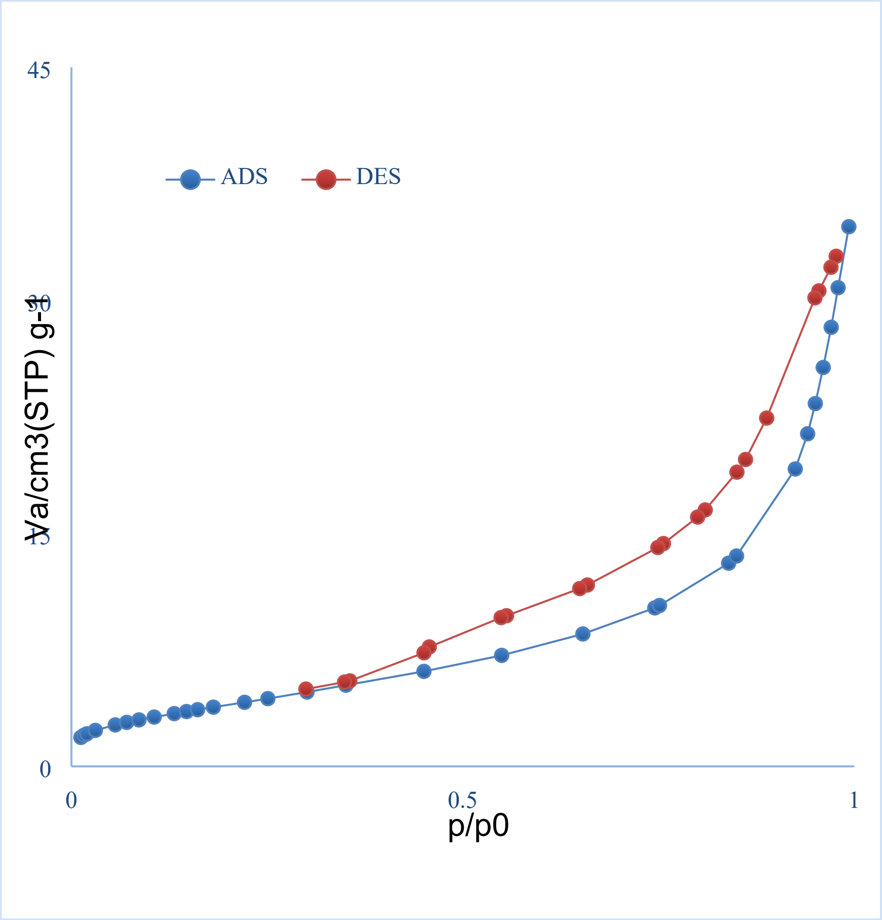

Fig. S3 Adsorption-desorption isotherm of $ZrFe_2O_4$ MNPs.

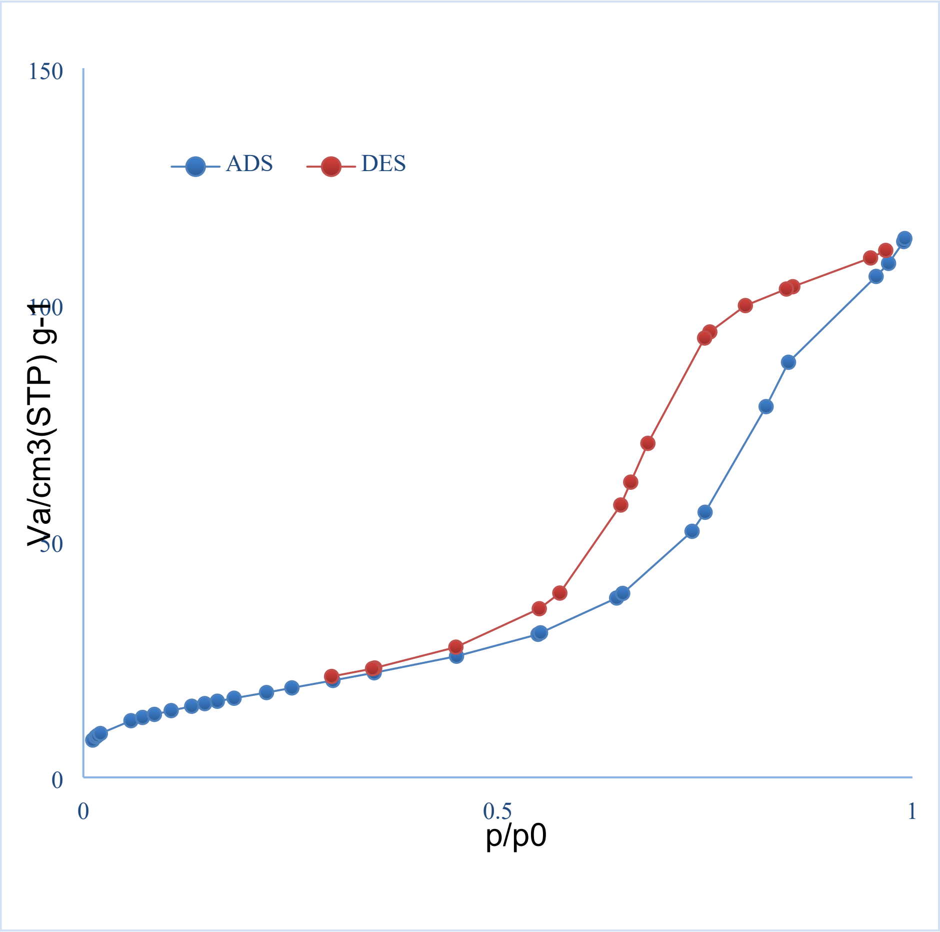

Fig. S4 Adsorption-desorption isotherm of $ZrFe_2O_4$@UiO-66-NH_2 nanocomposite.

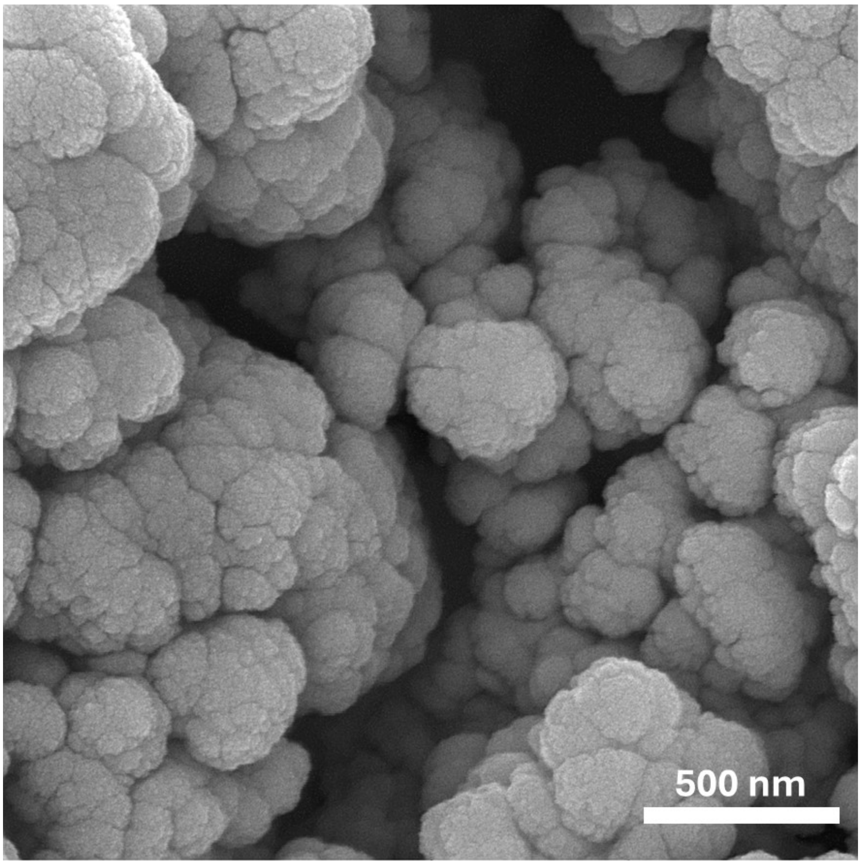

Fig. S5 SEM image of immobilized lipase on activated $ZrFe_2O_4$ nanoparticles.

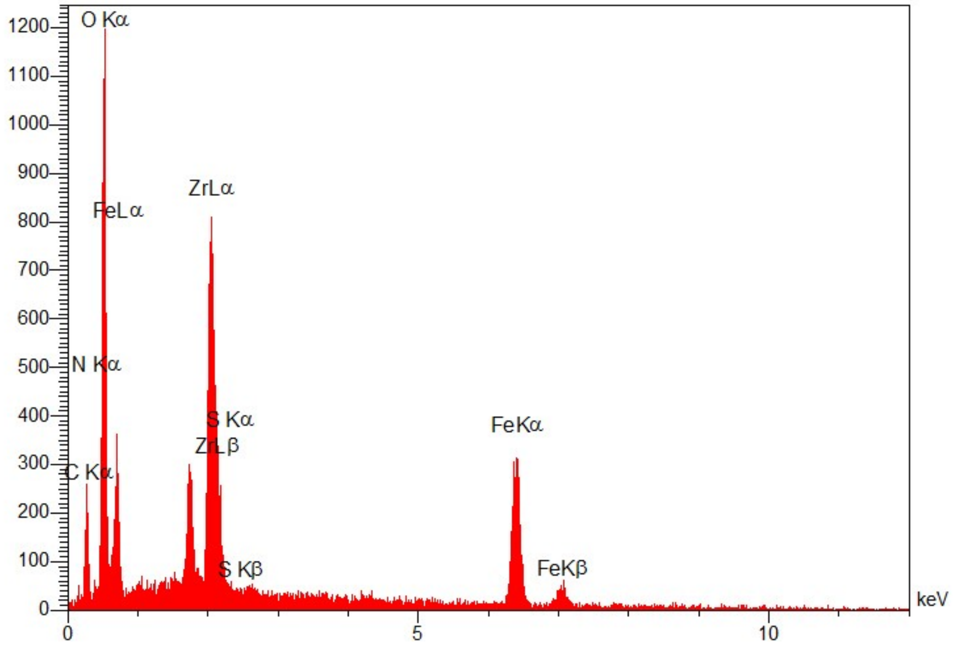

Fig. S6 Energy dispersive X-ray spectrum of immobilized lipase on activated $ZrFe_2O_4$ nanoparticles.

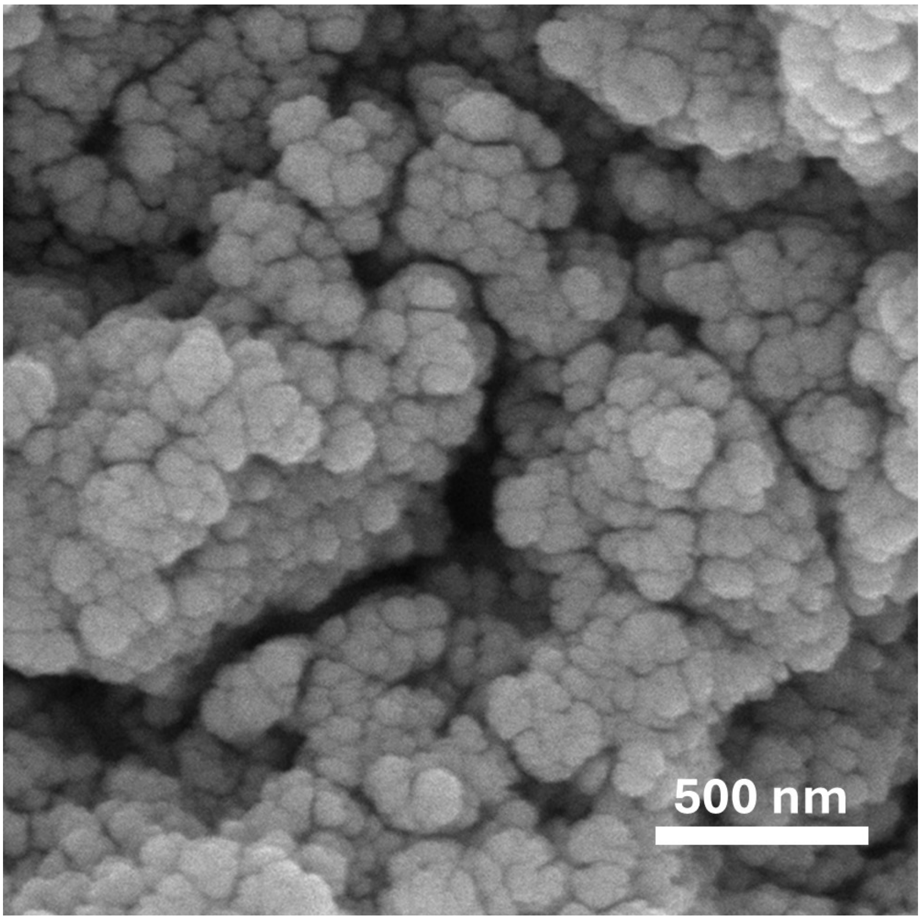

Fig. S7 SEM image of immobilized lipase on $ZrFe_2O_4$@UiO-66-NH_2.

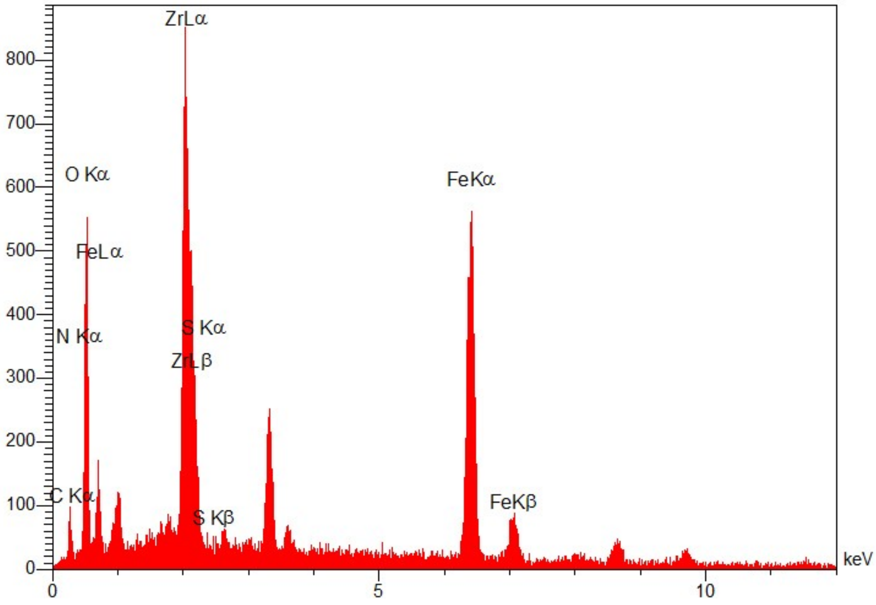

Fig. S8 Energy dispersive X-ray spectrum of immobilized lipase on $ZrFe_2O_4$@UiO-66-NH_2.

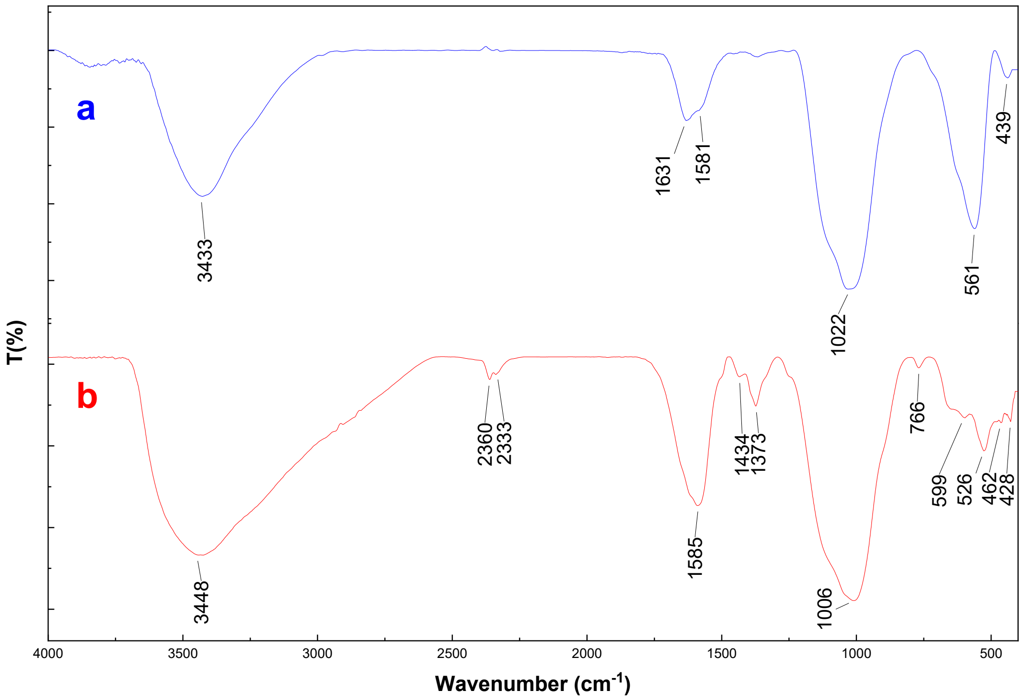

Fig. S9 FTIR spectra of the (a) immobilized lipase on the $ZrFe_2O_4$@UiO-66-NH_2 support in 50 mM phosphate buffer and (b) $ZrFe_2O_4$@UiO-66-NH_2 in phosphate buffer without enzyme.

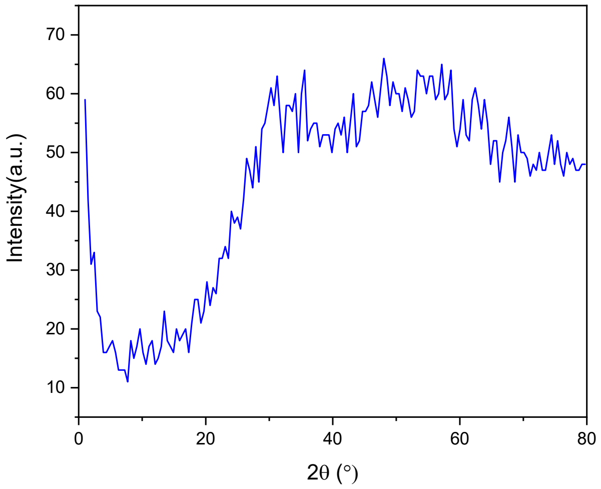

Fig S10 X-ray diffraction pattern of $ZrFe_2O_4$@UiO-66-NH_2 after immersion in phosphate buffer.

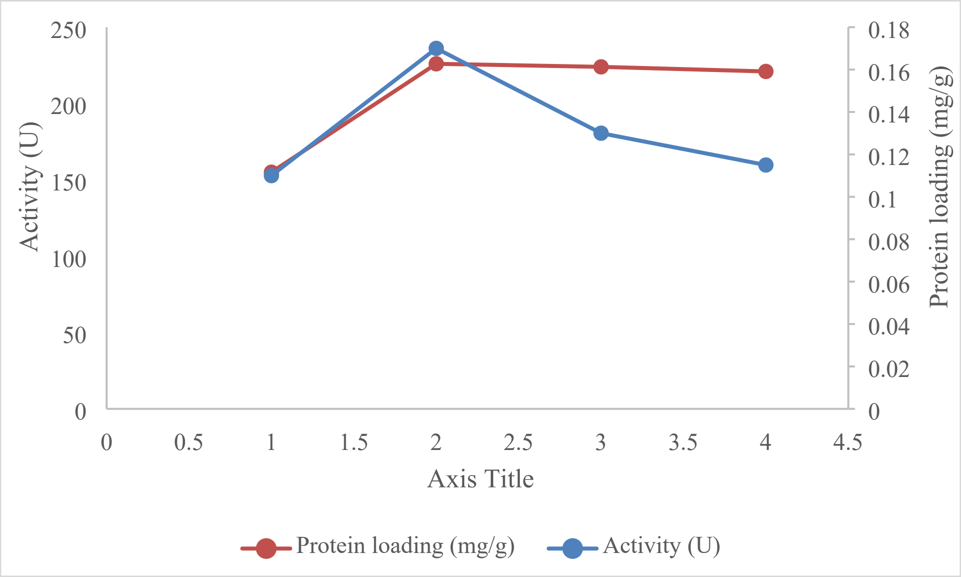

Fig. S11 Effect of cross-linking time on the specific activity of immobilized lipase on $ZrFe_2O_4$@UiO-66-NH_2 support.

Table 2. Immobilization yield, enzyme loading, and the immobilization efficiency of the biocatalysts. The data are expressed as mean ±SD (n =3).

**Parameter**	Immobilization yield (%) [a]		mg lipase/g support		Immobilization efficiency (IE, %) [b]	
**Method / Support**	*Precipitation-crosslinking*	*Covalent binding*	*Precipitation-crosslinking*	*Covalent binding*	*Precipitation-crosslinking*	*Covalent binding*
$ZrFe_2O_4$	62±0.82	73±0.5	260±3	305±1.6	40±1	65±3
$ZrFe_2O_4$@UiO-66-NH_2	54±0.5	60±0.47	226±2.4	254±2.9	60±2	80±1.6

[a] Immobilization yield is calculated as the percentage of enzyme molecules that are attached to the carrier.

[b] Immobilization efficiency is defined as the ratio of the specific activity of the immobilized enzyme to the specific activity of the soluble one.
